# Boundary-guided VMUNet for atrophic gastritis lesion segmentation in magnifying endoscopy with narrow-band imaging

**DOI:** 10.3389/fmed.2026.1871768

**Published:** 2026-08-19

**Authors:** Xiaoyu Chen, Kun Jiang, Xiufeng Su, Liyong Ma

**Affiliations:** 1 School of Information Science and Engineering, Harbin Institute of Technology, Weihai, Shandong, China; 2 Weihai Municipal Hospital, Cheeloo College of Medicine, Shandong University, Weihai, Shandong, China

**Keywords:** artificial intelligence, atrophic gastritis, lesion segmentation, magnifying endoscopy, narrow-band imaging, visual state-space model, weak boundary

## Abstract

**Background:**

Atrophic gastritis (AG) is a gastric precancerous condition, and objective delineation of lesion extent may support risk assessment, targeted biopsy, and standardized endoscopic documentation. Magnifying endoscopy with narrow-band imaging (ME-NBI) highlights mucosal and microvascular patterns, but automated segmentation remains difficult because AG lesions often show weak, gradual, and heterogeneous boundaries.

**Methods:**

We conducted a single-center retrospective study including 137 ME-NBI images of AG and 87 images of intestinal metaplasia (IM) from 25 subjects. Expert consensus masks were used as reference standards. We developed boundary-guided VMUNet (BG-VMUNet), a weak-boundary-aware segmentation framework combining a five-channel chromatic-textural input representation, visual state-space modeling, coordinate attention, and boundary-guided feature modulation. Model performance was evaluated using five-fold cross-validation and compared with representative segmentation baselines. Dice coefficient, intersection over union (IoU), precision, recall, and 95th percentile Hausdorff distance (HD95) were used to assess region overlap and boundary localization. An extended validation experiment was performed on IM segmentation.

**Results:**

In AG segmentation, BG-VMUNet achieved a Dice coefficient of 0.7228 ± 0.0504, IoU of 0.6079 ± 0.0592, precision of 0.7111 ± 0.0658, recall of 0.8266 ± 0.0265, and HD95 of 94.4665 ± 17.8695 pixels. The five-channel chromatic-textural representation and the boundary-guided architecture contributed separately: the representation provided a general, transferable benefit across backbones, whereas the boundary-guided components added further improvement on top of the representation. In a subject-level paired analysis after aggregating AG validation images within each subject, BG-VMUNet showed statistically significant improvements over the RGB VMUNet baseline in Dice, IoU, and precision after Holm correction, whereas the differences in recall and HD95 were favorable in direction but did not reach significance. In the IM extension task, BG-VMUNet showed numerically higher Dice, IoU, and precision and a lower HD95 than the baseline, with a slightly lower recall.

**Conclusion:**

BG-VMUNet provides a feasible weak-boundary-aware approach for automated AG lesion segmentation in ME-NBI images. By improving region-level delineation under challenging mucosal patterns, this framework may provide a technical basis for future quantitative assessment of gastric precancerous conditions, subject to multicenter external validation and explicit linkage to clinical endpoints. These findings are preliminary and exploratory.

## 1 Introduction

Gastric cancer remains a major global health burden, and its prevention depends not only on the detection of established malignancy but also on the timely recognition and management of epithelial precancerous conditions (1). Atrophic gastritis (AG) and intestinal metaplasia (IM) are key lesions in the Correa cascade of gastric carcinogenesis. Their clinical significance is not limited to binary presence or absence; rather, the extent, topographic distribution, and histological severity of atrophy and metaplasia are central to individualized risk assessment and surveillance planning (2–6). This concept is operationalized in the OLGA and OLGIM staging systems, which integrate lesion severity and anatomical distribution to identify patients at increased risk of gastric cancer (7, 8). Prospective evidence has further shown that more extensive and severe gastric IM is associated with a higher subsequent risk of gastric cancer (9). Therefore, objective region-level assessment of AG and IM on endoscopic images may provide clinically relevant inputs for lesion-burden estimation, longitudinal documentation, biopsy planning, and future risk modeling.

Magnifying endoscopy with narrow-band imaging (ME-NBI) enables enhanced visualization of gastric mucosal microsurface and microvascular patterns and has been increasingly used for the optical diagnosis of gastric precancerous conditions (10, 11). In clinical practice, endoscopic atrophic borders, mucosal pattern changes, and IM-related findings such as the light blue crest provide important visual cues for diagnosis and grading (10, 12). However, the interpretation of these findings remains influenced by operator experience, image quality, lesion location, and interobserver variability. Even for conventional endoscopic grading of gastric atrophy, systematic training can improve agreement but does not fully eliminate subjectivity (13). These limitations are particularly relevant when the clinical question shifts from image-level recognition to region-level delineation: the endoscopist must not only identify whether a lesion is present, but also determine where it begins, how far it extends, and whether its border can be consistently recorded.

Artificial intelligence (AI)-assisted digestive imaging has rapidly evolved from visual detection toward clinically oriented tasks such as quantitative evaluation, staging support, and individualized decision-making. In gastric precancerous disease, deep learning studies have reported encouraging results for the detection of precancerous conditions, real-time decision support across gastric carcinogenesis stages, semantic segmentation of gastric IM, and ME-NBI-based prediction of IM grading or high-risk OLGIM staging (14–17). Recent systematic evidence further indicates that AI-assisted endoscopy can achieve high pooled accuracy for gastric intestinal metaplasia detection, supporting its diagnostic potential in gastric precancerous conditions (18). Beyond gastric lesions, recent digestive imaging studies have also emphasized that imaging AI should be anchored to clinically meaningful endpoints, such as non-invasive molecular prediction in colorectal cancer or improved imaging-based staging for superficial gastrointestinal neoplasia (19, 20). These studies suggest that the value of AI in digestive diseases lies not merely in achieving higher numerical performance, but in converting image information into outputs that can assist diagnosis, staging, treatment selection, and follow-up management.

Despite these advances, pixel-level segmentation of AG regions in ME-NBI images remains underexplored compared with image-level classification or IM-focused tasks. This gap is clinically important because AG lesions often present as continuous or patchy mucosal changes with weak, gradual, and sometimes ill-defined boundaries. Unlike many polyp or tumor segmentation tasks in which the target may be more morphologically distinct from the surrounding tissue, AG in ME-NBI images is frequently characterized by subtle tonal shifts, heterogeneous mucosal texture, and cross-regional transitions in microsurface patterns. These imaging characteristics make lesion delineation challenging for both manual annotation and automated modeling. They also indicate that effective AG segmentation should incorporate sensitivity to weak boundary transitions, local texture heterogeneity, and non-local continuity of mucosal patterns, so that the resulting lesion maps can better reflect the region-level information needed for clinical assessment.

General medical image segmentation has been strongly influenced by encoder–decoder architectures such as U-Net and by robust self-configuring frameworks such as nnU-Net (21, 22). In parallel, pretraining, semi-supervised learning, and transformer-based architectures have been introduced to improve representation learning under limited annotation and complex anatomical contexts (23–25). More recently, state space models, including Mamba and visual Mamba variants, have attracted attention because they can model long-range dependencies with favorable computational efficiency (26, 27). Their integration into medical segmentation frameworks, such as U-Mamba and VM-UNet, suggests a promising direction for modeling spatially distributed lesion patterns (28, 29). However, ME-NBI-based AG segmentation requires more than generic long-range modeling. The model must also preserve subtle color information, capture local texture inhomogeneity, suppress highly textured background mucosa, and maintain sensitivity to ambiguous lesion borders. Boundary-aware learning and contour-sensitive evaluation have therefore become important considerations for segmentation tasks involving unclear margins (30–33), including boundary-aware transformer designs that adaptively emphasize ambiguous margins (34).

To address this clinically motivated imaging problem, we developed a boundary-guided visual state-space segmentation framework for AG lesion delineation in ME-NBI endoscopic images. The framework combines complementary image representations and boundary-sensitive contextual modeling according to the visual characteristics of AG. First, in addition to RGB information, hue and pixel inhomogeneity factor (PIF) channels are introduced to emphasize chromatic variation and local texture heterogeneity. Second, spatial attention is used to enhance lesion-related responses while reducing interference from non-lesion mucosal texture (35). Third, visual state-space modeling is adopted to capture cross-regional mucosal pattern continuity with efficient long-range dependency modeling (26–29). Finally, a boundary-guided modulation mechanism is incorporated to strengthen feature updating and fusion around weak transition zones, where AG borders are most likely to be ambiguous. This design aims to produce segmentation outputs that are not only numerically accurate but also clinically interpretable as region-level lesion maps for extent assessment.

In this study, we performed a single-center retrospective evaluation of the proposed framework using clinically acquired ME-NBI images of AG, with additional validation on IM as another representative gastric precancerous lesion. The study was organized to reflect a clinical translation pathway: comparison with representative segmentation baselines, progressive evaluation of each problem-driven design component, qualitative assessment of weak-boundary cases, and exploratory testing of cross-lesion transferability. Generative white-light-to-narrow-band style augmentation was investigated only as an auxiliary strategy for limited-sample learning, given that synthetic data may improve appearance diversity but should not replace validation on real clinical images (36, 37). Through this design, we aimed to determine whether weak-boundary-aware visual state-space segmentation can provide a feasible technical basis for objective delineation and extent quantification of gastric precancerous lesions in ME-NBI endoscopy.

## 2 Materials and methods

### 2.1 Study design, data source, and reference standard

This study was designed as a single-center retrospective imaging study to develop and evaluate an automated segmentation framework for region-level delineation of gastric precancerous lesions in magnifying endoscopy with ME-NBI. The primary task was AG lesion segmentation, because objective delineation of AG extent may provide image-derived support for early recognition of gastric precancerous mucosal changes, standardized endoscopic documentation, lesion-burden assessment, and future risk-oriented surveillance. IM, another important gastric precancerous lesion, was included as an extended validation task to examine whether the same weak-boundary-aware framework could be applied to a related ME-NBI segmentation scenario.

The ME-NBI images were retrospectively collected from routine clinical endoscopic examinations performed at Weihai Municipal Hospital, Cheeloo College of Medicine, Shandong University. Images were acquired using Olympus magnifying gastroscopes (GIF-H260Z and GIF-H290Z; Olympus Medical Systems, Tokyo, Japan) with an Olympus CV-290 video processor and an Olympus CLV-290SL light source. The images were exported at a resolution of 1920 × 1,080 pixels and were subsequently resized to 512 × 512 pixels for model training and evaluation. The study was approved by the Ethics Committee of Weihai Municipal Hospital, Cheeloo College of Medicine, Shandong University (Approval Code: 2020027; Approval Date: June 16, 2020). All images were de-identified before analysis. Owing to the retrospective nature of the study and the use of de-identified endoscopic images, the requirement for written informed consent was waived by the ethics committee.

Images were included if they met the following criteria: (1) the patient had undergone gastroscopy with ME-NBI image acquisition; (2) the image contained valid lesion-related regions corresponding to AG or IM; and (3) the image had sufficient resolution, clarity, and exposure quality for lesion annotation and model evaluation. Images were excluded if they were severely blurred, out of focus, overexposed, underexposed, affected by obvious motion artifacts, mucus obstruction, instrument occlusion, or severe reflection. Duplicate or near-duplicate frames of the same lesion were removed, and only the image with better visual quality was retained. Images with incomplete annotation information or without expert consensus were also excluded.

After screening, 137 AG ME-NBI images were included for the main segmentation task, and 87 IM ME-NBI images were included for extended validation. The included images were obtained from 25 subjects. When multiple images were available from the same patient, patient-level allocation was controlled during data partitioning to reduce information leakage between training and validation sets.

Lesion labels were determined through a comprehensive review of endoscopic findings, pathological results, and clinical records. Region-level annotations were performed on the original ME-NBI images using ITK-SNAP by two endoscopists with experience in gastrointestinal endoscopy. The objective was to delineate the entire visible lesion extent rather than a single contour line. Each lesion was traced by manual free-form delineation as one or more closed regions, which were then filled to generate a binary mask in which lesion pixels were labeled as foreground and all remaining pixels as background. The annotation protocol did not restrict each image to a single convex polygon; an image was allowed to contain multiple disjoint closed regions and internal holes (i.e., enclosed non-lesion mucosa), so that irregular, patchy, or spatially discontinuous AG/IM regions could be annotated in accordance with their actual endoscopic appearance. For lesions with clear margins, the visible border was traced directly. For lesions with weak, gradual, or heterogeneous boundaries, the annotators first identified the visually recognizable lesion core and then progressively extended the annotation into adjacent transition zones in which lesion-related ME-NBI features (mucosal microsurface structure, microvascular morphology, the atrophic border, gradual tonal or chromatic transition, and the light blue crest for IM when present) remained evident; adjacent mucosa without consistent lesion-related findings was deliberately excluded to avoid over-expansion of the mask. In cases of disagreement, three senior experts reviewed the images and reached a consensus, which was used as the reference-standard mask for model training and evaluation. Because the reference standard was established by expert consensus, the consensus masks were used directly for model training and evaluation, and a quantitative inter-observer agreement analysis was not performed; the residual subjectivity of manual delineation, particularly in weak-boundary regions, is acknowledged in the Limitations.

A single-class segmentation setting was adopted for each task. In the original color annotations, the red mask corresponded to AG, whereas the green mask corresponded to IM. The network output was a single-channel lesion probability map, which was converted into a binary mask after Sigmoid activation and thresholding at 0.5. The resulting mask was regarded as a region-level imaging output rather than a diagnostic label alone. It could be overlaid on the original ME-NBI image to visualize lesion distribution and could, in future work and subject to further validation, serve as a basis for lesion extent quantification and morphological feature extraction.

### 2.2 Overall framework and five-channel image representation

**Figure 1:**
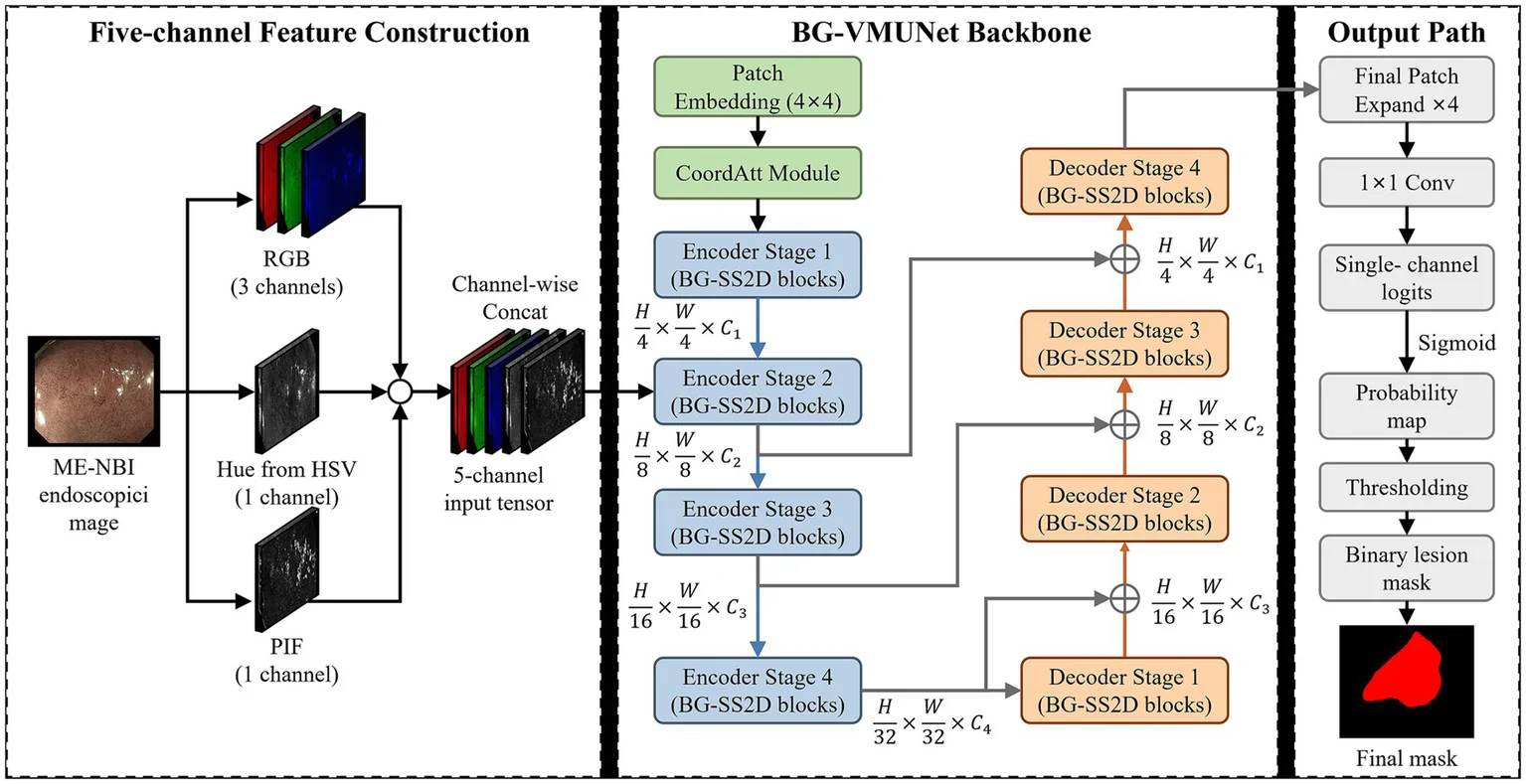
Schematic overview of the proposed BG-VMUNet framework for atrophic gastritis lesion segmentation in ME-NBI images. The input image is first converted into a five-channel representation composed of RGB, hue, and PIF channels, and then processed by the BG-VMUNet backbone to generate a single-channel lesion probability map and the final binary segmentation mask.

The overall workflow of the proposed framework is shown in Figure 1. The methodological design was guided by the characteristic appearance of AG in ME-NBI images, namely subtle chromatic changes, heterogeneous mucosal texture, gradual lesion transitions, and weak or ill-defined borders. Instead of relying only on the original RGB image, the proposed framework first constructs a five-channel representation to strengthen lesion-related low-level information. The input is then processed by a boundary-guided visual state-space segmentation network, referred to as BG-VMUNet, to generate a single-channel lesion probability map and the final binary lesion mask.

All images were resized to 512 × 512 pixels before being input into the network. The five-channel chromatic-textural representation is one of the explicitly stated contributions of this work rather than an undisclosed input advantage. To evaluate the backbone and the architectural design fairly while separating their effect from the input representation, the comparison experiments were organized in two stages. First, representative segmentation models were compared under a common three-channel RGB input to provide a reference baseline and to identify a suitable backbone architecture. Second, all comparison models were re-trained under the identical five-channel input, using exactly the same data partitions, preprocessing, augmentation, loss, optimizer, and evaluation protocol, so that the contribution of the backbone and architecture could be assessed independently of the input representation. For the proposed framework, the same five-channel representation was constructed by concatenating the original RGB channels, the Hue channel, and the pixel inhomogeneity factor (PIF) channel.

**Figure 2:**
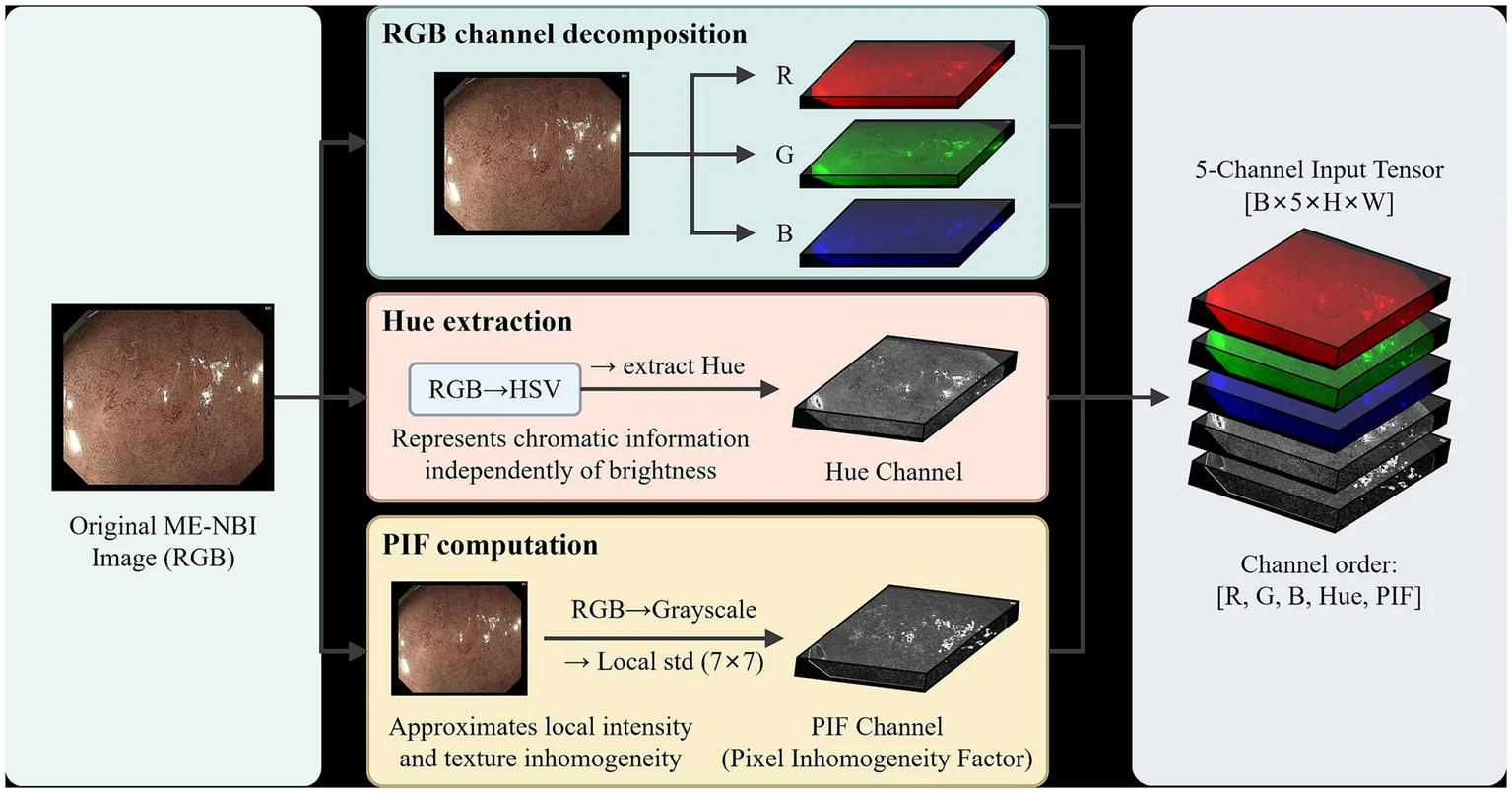
Construction process of the five-channel input representation. The original ME-NBI image is preserved as RGB channels, while the Hue channel is extracted from the HSV color space and the PIF channel is computed from local grayscale intensity variation. These channels are concatenated to form the final five-channel input tensor.

The construction of the five-channel representation is illustrated in Figure 2. The RGB image provides basic color and texture information. The Hue channel was extracted after conversion from RGB to HSV color space. Hue describes chromatic information relatively independent of brightness and was introduced to enhance sensitivity to subtle tonal changes around AG lesions. To further describe local mucosal texture heterogeneity, the PIF channel was computed from the grayscale luminance image. Specifically, for each pixel, the local standard deviation within a 7×7 neighborhood was calculated:


$$\mathrm{PIF}(x,y)=\sqrt{E_{\Omega }(I_{g}^{2})-{\left[E_{\Omega }(I_{g})\right]}^{2}}$$

where Ig denotes the grayscale luminance image, and *Ω* denotes the local neighborhood centered at pixel (x, y).

The final five-channel input tensor was defined as:


$$X_{5c}=\text{Concat}(R,G,B,\mathrm{Hue},\mathrm{PIF})$$

This representation was used to provide complementary visual cues: RGB for general appearance, Hue for chromatic transition, and PIF for local texture or luminance inhomogeneity. This design is particularly relevant for AG segmentation because AG borders in ME-NBI images are often gradual rather than sharply demarcated, and lesion-related information may be distributed across both color and texture changes.

**Figure 3:**
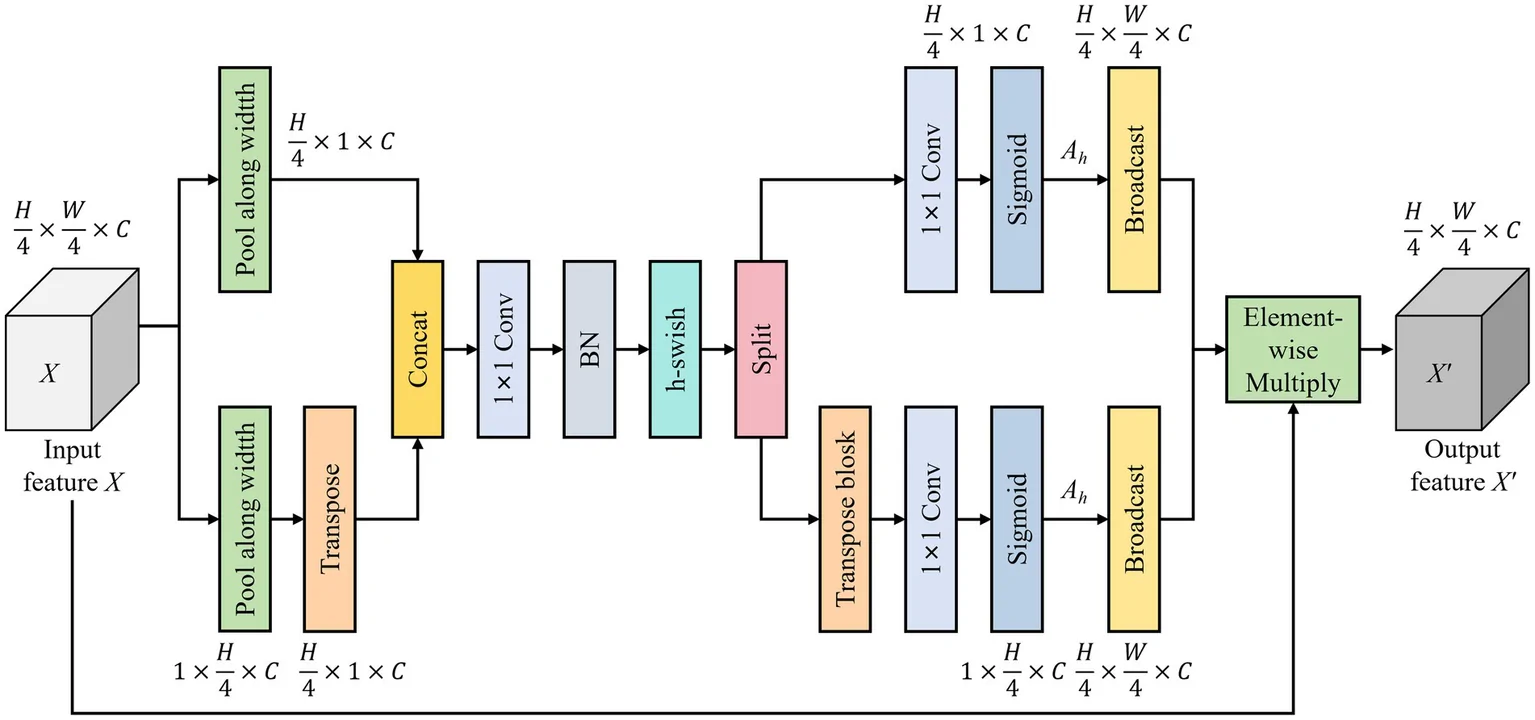
Structure of the CoordAtt module used in BG-VMUNet. The module aggregates spatial information along the horizontal and vertical directions, generates direction-aware attention weights, and recalibrates the input feature maps to enhance lesion-related spatial responses.

BG-VMUNet was built upon the VMUNet architecture while being adapted to the weak-boundary and heterogeneous-texture characteristics of ME-NBI images. The network retained a hierarchical encoder–decoder structure and produced a single-channel lesion probability map. The encoder and decoder depths were set to [2, 2, 2, 2] and [2, 2, 2, 1], respectively, and the stochastic depth rate was set to 0.2. After patch embedding, coordinate attention was introduced at the shallow feature stage to preserve positional information and recalibrate lesion-related responses along horizontal and vertical directions (Figure 3). In ME-NBI images, this operation was used to enhance responses to mucosal texture transitions and lesion boundary regions while suppressing interference from highly textured non-lesion mucosa.

### 2.3 Boundary-guided visual state-space modeling

The core contextual modeling unit of BG-VMUNet was a two-dimensional selective scan module. This component was used to capture cross-regional mucosal pattern continuity, which is important because AG lesions often extend gradually across the mucosal surface rather than appearing as compact objects with sharp edges. Let the input feature of the selective scan module be:


$$Χ\in {\mathbb{R}}^{B\times H\times W\times C}$$

where B , H , W , and C denote batch size, height, width, and channel number, respectively. The input feature was first mapped into the latent space and split into two branches:


$$\left[X_{v},Z]=\text{Linear}(Χ\right)$$

where $X_{v}$ was used for state-space contextual modeling, and Z served as the gating branch. The modeling branch was then rearranged and processed by depthwise convolution and SiLU activation to obtain intermediate spatial features:


$$X_{c}=\text{SiLU}(\text{DWConv}(X_{v}))$$

To model mucosal patterns that may extend in different spatial directions, $X_{c}$ was flattened into four directional sequences: horizontal forward, vertical forward, horizontal reverse, and vertical reverse. These sequences were denoted as:


$${\left\{S^{\left(k\right)}\right\}}_{k=1}^{4},S^{\left(k\right)}\in {\mathbb{R}}^{B\times D\times L},L=H\times W$$

where D denotes the expanded hidden dimension and L denotes the sequence length. Each directional sequence was processed by a selective scan branch. After restoring the reverse and column-major sequences to the original spatial arrangement, the directional outputs were aggregated:


$$Y_{\text{scan}}=\sum_{k=1}^{4}{ℛ}_{k}(Y^{\left(k\right)})$$

where $Y^{\left(k\right)}$ denotes the output of the k -th directional scan, and ${ℛ}_{k}(\cdot )$ denotes restoration to the original spatial orientation.

**Figure 4:**
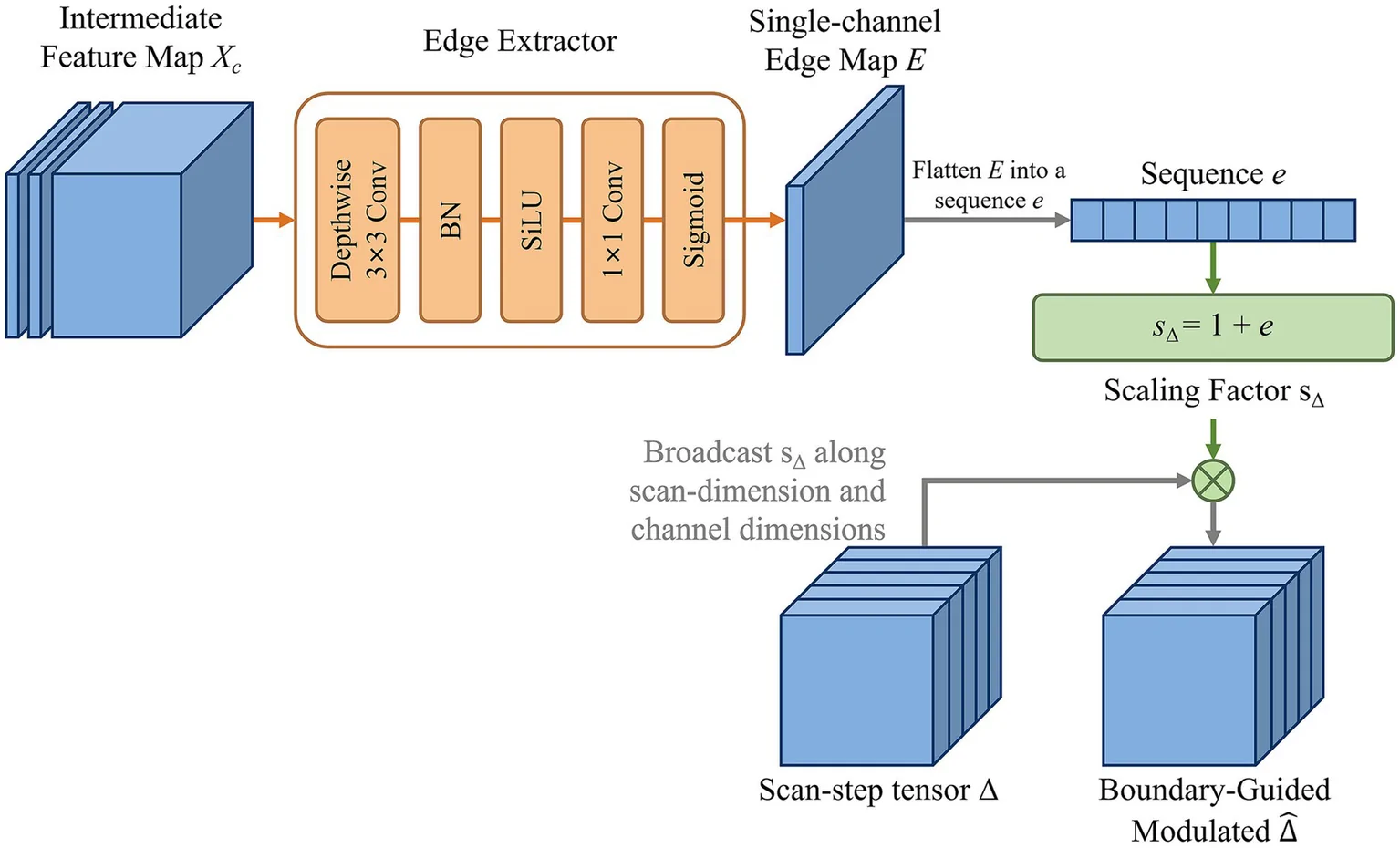
Schematic illustration of the boundary-guided modulation process in the SS2D module. Boundary responses are extracted from intermediate features and used to modulate the selective scan step size, allowing the model to increase sensitivity around weak and gradual lesion boundaries.

To improve sensitivity to weak and gradual lesion borders, boundary-guided modulation was incorporated into the visual state-space block (Figure 4). Unlike strategies that add boundary constraints only at the output end, this mechanism extracts boundary responses from intermediate features and uses them to modulate both contextual feature propagation and post-scan feature fusion. A lightweight edge extraction branch $f_{\text{edge}}(\cdot )$ , consisting of depthwise convolution, normalization, activation, and pointwise convolution, was applied to $X_{c}$ . The boundary response map was defined as:


$$e=\text{Flatten}(E),e\in {\mathbb{R}}^{B\times 1\times L}$$

A dynamic scaling factor was generated as follows:


$$s_{\Delta }=1+e$$

where e is a sigmoid-bounded edge response with e ∈ [0,1]; consequently the scaling factor is bounded as $\;s_{\Delta }\in [1,2].$ Given the original step-size term *Δ*, the boundary-guided step size was computed as:


$$\Delta^{\prime }=\Delta ⊙s_{\Delta }$$

where ⊙ denotes element-wise multiplication, and $s_{\Delta }$ is broadcast along the channel and scan-direction dimensions.

This asymmetric, amplify-only design is intentional and conservative for three reasons. First, in homogeneous intra-lesion or background regions the edge response is low ( e≈0 ), so $s_{\Delta }\approx 1$ and the modulated step size approaches the original *Δ*; the mechanism therefore largely preserves the original scan behavior in such regions rather than altering it. Second, near weak transition zones ( e→1 ) the effective step size at most doubles, allowing a bounded adjustment of the selective-scan state around mucosal transition regions while remaining bounded and stable. Third, allowing suppression $\left(s_{\Delta }<1\right)$ would attenuate state propagation precisely in the weak-boundary regions that the design intends to emphasize, and an unbounded multiplier would risk unstable updates; the bounded amplify-only form avoids both. This design allows the state update process to receive stronger modulation near boundary-like transition regions while maintaining smoother propagation in relatively homogeneous intra-lesion or background regions.

After directional scanning, the boundary response was further used for boundary-enhanced feature fusion, forming the second part of the boundary-guidance mechanism. The boundary map was spatially aligned and expanded to match the feature dimension, denoted as E’. The fused feature was computed as:


$$Y_{\text{fuse}}=Y_{\text{scan}}⊙(1+E^{\prime })+{\text{Conv}}_{1\times 1}(E^{\prime })$$

The first term performs multiplicative boundary gating that enhances boundary-neighborhood responses, whereas the second term provides additive residual boundary compensation. The fused feature was subsequently processed by normalization, gating with Z, and linear projection to generate the output of the block. Through step-size modulation and post-scan fusion, the model was encouraged to treat weak transition zones differently from relatively uniform mucosal regions, which is consistent with the visual challenge of AG delineation in ME-NBI images.

### 2.4 Training, validation, and descriptive performance evaluation

For the main AG task, a predefined five-fold cross-validation protocol was used. The training and validation assignments were fixed across experiments to ensure fair comparison among models and configurations. Patient-level allocation was controlled when multiple images originated from the same subject. For the IM extension experiment, the same evaluation workflow was applied to examine the applicability of the framework in another gastric precancerous lesion segmentation scenario.

The experimental design followed a stepwise validation strategy. First, representative segmentation models were compared under a common three-channel RGB input and training protocol to identify a suitable baseline architecture for ME-NBI AG segmentation, and all comparison models were additionally re-trained under the identical five-channel input to separate the contribution of the input representation from that of the backbone and architecture. The comparison models included U-Net, U-Net++, FCN, SegNet, PSPNet, FPN, Attention U-Net, DeepLabV3+, SegNeXt, Swin-Unet, nnU-Net, and VMUNet. Second, progressive validation was performed to evaluate the contribution of each design element, including five-channel input representation, coordinate attention, visual state-space branch adaptation, and boundary-guided modulation. Third, the final framework was applied to IM lesion segmentation to explore cross-lesion transferability within gastric precancerous lesions.

During training, standard image augmentation was applied to improve robustness under limited-sample conditions. The augmentation strategy included translation, scaling, rotation, flipping, and size normalization. If the runtime environment did not support the complete augmentation library, random horizontal flipping, random vertical flipping, and resizing were used as a simplified augmentation scheme. During validation, only size normalization was performed, and no random augmentation was applied.

An auxiliary generative augmentation experiment was also performed to explore limited-sample learning. Synthetic training data were generated using a CycleGAN-based unpaired image-to-image translation framework, following the white-light-to-narrow-band style-transfer concept reported for endoscopic imaging. White-light endoscopic images were used as the source domain, and real ME-NBI images were used as the target domain, so that the visual appearance of white-light images could be translated into an NBI-like style to increase appearance diversity under limited-sample conditions. The CycleGAN model was implemented in PyTorch and trained using 141 white-light images and 159 NBI images at a resolution of 256 × 256 pixels for 200 epochs. After training, 38 white-light AG endoscopic images from Weihai Municipal Hospital were translated into NBI-style images for this auxiliary strategy. Each synthetic image was visually reviewed for quality, and images with obvious artifacts, severe structural deformation, or unrealistic color appearance were excluded. The masks from the original white-light images were not reused; instead, the retained NBI-style synthetic images were manually annotated using the same region-level annotation protocol applied to the real ME-NBI images, and these masks were used as supervision labels. The retained synthetic images and their newly annotated masks were introduced only into the corresponding training folds and were never placed in any validation fold; therefore, all validation results were evaluated exclusively on real ME-NBI images. This experiment was treated as an auxiliary and exploratory augmentation strategy for limited-sample learning rather than as primary evidence of clinical effectiveness, and it was reported separately from the main real-image validation results.

All experiments used the Adam optimizer with an initial learning rate of 0.0001. The batch size was set to 10, and the number of training epochs was set to 500. The loss function combined binary cross-entropy with logits loss and Dice loss with equal weights:


$$L=0.5L_{\mathrm{BCE}}+0.5L_{\text{Dice}}$$

During inference, the network output was passed through a Sigmoid activation function, and a fixed threshold of 0.5 was used to convert the probability map into a binary lesion mask.

To improve reproducibility and ensure fair comparison, all models were trained and validated using the same data partitions, preprocessing pipeline, augmentation strategy, loss function, optimizer, inference threshold, and evaluation metrics. The five-fold training and validation assignments were fixed across all baseline comparisons and ablation experiments. When multiple images originated from the same patient, patient-level allocation was controlled to reduce information leakage between the training and validation sets. No random augmentation was applied during validation, and all validation results were calculated on real ME-NBI images after resizing to a unified spatial resolution.

Model performance was evaluated from two complementary perspectives: region overlap quality and boundary localization quality. Dice coefficient and IoU were used to quantify the agreement between predicted and reference lesion regions. Precision and Recall were used to evaluate false-positive control and lesion coverage, respectively. The 95th percentile HD95 was used to assess boundary deviation between the predicted and reference contours. HD95 was calculated as the 95th percentile of the bidirectional surface distances between the predicted mask and the reference mask, thereby reducing the influence of extreme outlier boundary points compared with the maximum Hausdorff distance. Because all images were resized to 512 × 512 before model input and evaluation, HD95 was reported in pixels, with lower values indicating better boundary localization.


$$\text{Dice}=\frac{2\mathrm{TP}}{2\mathrm{TP}+\mathrm{FP}+\mathrm{FN}}$$

$$\mathrm{IoU}=\frac{\mathrm{TP}}{\mathrm{TP}+\mathrm{FP}+\mathrm{FN}}$$

$$\text{Precision}=\frac{\mathrm{TP}}{\mathrm{TP}+\mathrm{FP}}$$

$$\text{Recall}=\frac{\mathrm{TP}}{\mathrm{TP}+\mathrm{FN}}$$

where true-positive, false-positive, and false-negative pixels were denoted as TP , FP , and FN , respectively. Dice was used as the primary criterion for optimal model selection in each fold, because region overlap is central to lesion extent quantification. IoU, Precision, Recall, and HD95 were reported together to characterize the balance among lesion coverage, false-positive control, and boundary adherence. Quantitative results were summarized as mean ± standard deviation across five folds.

In addition, qualitative visualization was performed using representative validation cases to compare the original image, reference annotation, baseline prediction, and final model prediction. Special attention was paid to weak-boundary regions, heterogeneous mucosal texture, local false-positive expansion, and missed low-contrast lesion areas, because these conditions are clinically relevant to objective lesion extent assessment in ME-NBI endoscopy.

### 2.5 Statistical analysis

In addition to the descriptive mean ± standard deviation summaries, paired statistical analysis was performed to assess whether the observed differences between models were supported by the evaluation data. Because multiple images could originate from the same subject, the paired statistical analysis was conducted after subject-level aggregation rather than treating each image as an independent statistical unit. Specifically, for each subject, metric values were first averaged across all AG validation images from that subject, and paired differences between BG-VMUNet and each comparator were then computed at the subject level. This design preserved the within-subject correspondence between paired model predictions while reducing the risk of overestimating statistical evidence from correlated images.

For each metric, subject-level paired differences between BG-VMUNet and the comparator were computed. The 95% confidence interval of the paired mean difference was estimated by bootstrap resampling with 10,000 bootstrap samples. Raw *p* values were obtained using a two-sided paired permutation test based on random sign-flipping of the subject-level paired differences with 10,000 permutations. To account for multiple comparisons, Holm correction was applied across the five metrics within each paired comparison, and corrected values are reported as Holm-adjusted *p* values. A two-sided significance level of 0.05 was used.

## 3 Results

### 3.1 Baseline comparison for AG lesion segmentation

To determine an appropriate foundation for region-level delineation of AG lesions in ME-NBI images, representative segmentation models were first compared under the same three-channel RGB input setting and training protocol. As shown in Table 1, VMUNet achieved the best overall performance among the evaluated models under RGB input, with a Dice of 0.6964, IoU of 0.5811, Precision of 0.6788, and HD95 of 101.3377. Although some models showed slightly higher Recall, their region-overlap and boundary-localization metrics were inferior to those of VMUNet. This result suggests that visual state-space modeling is suitable for ME-NBI AG segmentation, where lesion regions often show gradual transitions and cross-regional mucosal pattern continuity rather than sharply demarcated object boundaries.

**Table 1:** Comparison of segmentation performance across different models on the AG task under three-channel RGB input.

Model (RGB)	Dice(↑)	IoU(↑)	Precision(↑)	Recall(↑)	HD95(↓)
U-Net	0.6920	0.5700	0.6675	0.8285	106.9129
U-Net++	0.6838	0.5624	0.6583	0.8219	109.7796
FPN	0.6819	0.5634	0.6588	0.8318	107.4061
PSPNet	0.6749	0.5524	0.6720	0.7948	104.1880
nnU-Net	0.6809	0.5565	0.6447	**0.8455**	121.8906
Attention U-Net	0.6798	0.5608	0.6725	0.8010	108.4293
FCN	0.6748	0.5571	0.6482	0.8191	112.5154
SegNet	0.6716	0.5491	0.6603	0.8047	111.0578
DeepLabV3+	0.6687	0.5492	0.6510	0.8071	107.9655
SegNeXt	0.6534	0.5309	0.6191	0.8256	115.7640
Swin-Unet	0.6190	0.4941	0.5675	0.8272	120.3775
**VMUNet**	**0.6964**	**0.5811**	**0.6788**	0.8149	**101.3377**

All models are evaluated under the same training protocol. Bold text indicates the best value for each metric.

Because the five-channel chromatic-textural representation is one of the contributions of this work, and because comparing a five-channel model against three-channel baselines would confound input representation with architecture, all twelve comparison models were re-trained under the identical five-channel input using the same data partitions, preprocessing, augmentation, loss, optimizer, and evaluation protocol. The results are summarized in Table 2, with the RGB results in Table 1 retained as a reference. Three observations can be made. First, the five-channel representation improved every backbone relative to its RGB counterpart (for example, U-Net from 0.6920 to 0.7076 and VMUNet from 0.6964 to 0.7124 in Dice), indicating a general and transferable benefit of the chromatic-textural representation rather than a model-specific effect. Second, under the identical five-channel input, VMUNet remained the strongest backbone on Dice (0.7124), IoU (0.5986), Precision (0.7077), and HD95 (97.4331), with only Recall being higher for nnU-Net (0.8477), reproducing the ranking observed under RGB input. Third, the full BG-VMUNet (Dice 0.7228) further improved over the five-channel VMUNet, indicating that the boundary-guided components added value on top of the chromatic-textural representation. Accordingly, the improvement of BG-VMUNet should be attributed separately to the chromatic-textural representation and to the boundary-guided architecture, rather than to architecture alone. VMUNet was therefore selected as the backbone for subsequent weak-boundary-aware refinement.

**Table 2:** Comparison of segmentation performance across different models on the AG task under the identical five-channel input.

Model (5-channel)	Dice(↑)	IoU(↑)	Precision(↑)	Recall(↑)	HD95(↓)
U-Net	0.7076	0.5901	0.6765	0.8302	99.9238
U-Net++	0.6901	0.5688	0.6832	0.8301	105.7268
FPN	0.6876	0.5710	0.6918	0.8320	102.2033
PSPNet	0.6823	0.5625	0.7012	0.8086	100.5340
nnU-Net	0.6887	0.5643	0.6754	**0.8477**	116.4502
Attention U-Net	0.6899	0.5669	0.6935	0.8119	105.2913
FCN	0.6812	0.5642	0.6731	0.8214	109.2564
SegNet	0.6786	0.5569	0.6915	0.8098	109.9263
DeepLabV3+	0.6754	0.5561	0.6811	0.8171	105.2831
SegNeXt	0.6670	0.5380	0.6403	0.8236	111.2846
Swin-Unet	0.6310	0.5096	0.5382	0.8329	117.4985
**VMUNet**	**0.7124**	**0.5986**	**0.7077**	0.8115	**97.4331**

All models are evaluated using the same data partitions, preprocessing, augmentation, loss, optimizer, and evaluation protocol. Bold text indicates the best value for each metric.

### 3.2 Progressive validation of the weak-boundary-aware framework

The progressive validation results are summarized in Table 3. Compared with the RGB-input VMUNet baseline, introducing the five-channel representation improved Dice from 0.6964 to 0.7124, IoU from 0.5811 to 0.5986, and Precision from 0.6788 to 0.7077, while reducing HD95 from 101.3377 to 97.4331. This improvement indicates that the additional Hue and PIF channels provided useful complementary information for AG regions, especially in images where lesion-related cues were distributed across subtle tonal variation and local texture heterogeneity. Importantly, the improvement was not achieved by simply expanding the predicted lesion area, because Recall remained similar between the RGB and five-channel settings.

**Table 3:** Progressive ablation results from the RGB VMUNet baseline to the final BG-VMUNet model on the AG task.

Model stage	Dice(↑)	IoU(↑)	Precision(↑)	Recall(↑)	HD95(↓)
VMUNet (RGB)	0.6964 ± 0.0699	0.5811 ± 0.0730	0.6788 ± 0.0988	0.8149 ± 0.0496	101.3377 ± 18.5561
VMUNet(5-channel)	0.7124 ± 0.0590	0.5986 ± 0.0653	0.7077 ± 0.0821	0.8115 ± 0.0566	97.4331 ± 21.7233
VMUNet + CoordAtt	0.7168 ± 0.0628	0.6004 ± 0.0691	**0.7196 ± 0.0739**	0.7919 ± 0.0333	94.6172 ± 15.0094
VMUNet + CoordAtt + modified scan branch	0.7188 ± 0.0630	0.6032 ± 0.0732	0.7063 ± 0.0784	0.8266 ± 0.0371	96.3479 ± 20.1167
**BG-VMUNet(final)**	**0.7228 ± 0.0504**	**0.6079 ± 0.0592**	0.7111 ± 0.0658	**0.8266 ± 0.0265**	**94.4665 ± 17.8695**

Results are reported as mean ± standard deviation across five-fold cross-validation. Bold text indicates the best value for each metric.

After incorporating coordinate attention, the model showed a further increase in Dice and IoU, with improvements in Precision and HD95. This pattern indicates better suppression of non-lesion mucosal responses and improved adherence to lesion-related regions. The subsequent visual state-space branch adaptation increased Recall to 0.8266, suggesting improved lesion coverage and continuity across spatially distributed mucosal changes. However, this stage also showed a slight decrease in Precision and an increase in HD95, indicating that stronger contextual aggregation alone could introduce local overextension near ambiguous borders.

The final BG-VMUNet achieved the best overall balance among lesion coverage, region overlap, and boundary localization. Compared with the RGB VMUNet baseline, the final model showed numerically higher Dice, IoU, Precision, and Recall and a lower HD95; the statistical support for these differences is reported in the following subsection. These results indicate that the proposed framework was associated with improvements in both pixel-wise overlap and contour reliability. This balance is relevant because lesion extent assessment requires both adequate coverage of the suspected AG region and avoidance of excessive expansion into surrounding textured mucosa.

### 3.3 Statistical testing

The paired statistical results are summarized in Table 4. For the primary comparison between BG-VMUNet and the RGB VMUNet baseline after subject-level aggregation, the improvements in Dice, IoU, and Precision remained statistically significant after Holm correction (Holm-adjusted *p* = 0.032, 0.037, and 0.029, respectively). The differences in Recall and HD95 were favorable in direction but did not reach significance after correction (Holm-adjusted *p* = 0.444 and 0.281, respectively). In contrast, the increments between adjacent ablation stages were not statistically significant after Holm correction: BG-VMUNet relative to the five-channel VMUNet, and relative to the immediately preceding stage (VMUNet + CoordAtt + modified scan branch), yielded Holm-adjusted *p* values of approximately 0.9 and 1.0 across all metrics. Accordingly, the statistically supported claim is that the complete weak-boundary-aware framework outperforms the RGB baseline on overlap-based metrics, whereas the per-stage increments are numerically higher and directionally consistent but not statistically significant after Holm correction under the present sample size.

**Table 4:** Subject-level paired statistical comparison between BG-VMUNet and selected models in the AG task.

Comparison	Metric	mean_diff	95% CI	p_raw	p_holm
bg_vmunet: RGB VMUNet baseline	Dice	0.0264	[0.0077, 0.0463]	0.0080	0.0320
	IoU	0.0268	[0.0071, 0.0480]	0.0123	0.0369
	Precision	0.0323	[0.0091, 0.0568]	0.0058	0.0290
	Recall	0.0117	[−0.0165, 0.0394]	0.4435	0.4435
	HD95	−6.8712	[−15.8168, 2.0521]	0.1404	0.2808
bg_vmunet: Five-channel VMUNet	Dice	0.0104	[−0.0041, 0.0256]	0.1778	0.8889
	IoU	0.0093	[−0.0066, 0.0256]	0.2576	0.8889
	Precision	0.0035	[−0.0120, 0.0188]	0.6598	0.8889
	Recall	0.0150	[−0.0086, 0.0390]	0.2155	0.8889
	HD95	−2.9666	[−9.6934, 3.2657]	0.3804	0.8889
bg_vmunet: CoordAtt + modified scan branch	Dice	0.0040	[−0.0105, 0.0176]	0.5790	1.0000
	IoU	0.0047	[−0.0117, 0.0209]	0.5903	1.0000
	Precision	0.0049	[−0.0111, 0.0215]	0.5556	1.0000
	Recall	−0.0001	[−0.0251, 0.0216]	0.9103	1.0000
	HD95	−1.8814	[−8.6368, 4.9859]	0.6063	1.0000

For each subject, metric values were first averaged across all AG images from that subject, and paired differences were then computed between BG-VMUNet and each comparator. Mean difference is calculated as BG-VMUNet minus the comparator. The 95% CI was estimated by bootstrap resampling with 10,000 resamples; raw *p* values were obtained using a two-sided paired permutation test with 10,000 sign-flips; Holm-adjusted *p* values were corrected across the five metrics within each paired comparison.

### 3.4 Component-wise ablation of the boundary-guidance mechanism

To attribute the contributions of the two parts of the boundary-guidance mechanism, a finer-grained ablation was performed, comparing the no-boundary-guidance model (VMUNet + CoordAtt + modified scan branch), step-size modulation only (*Δ* modulation only), post-scan fusion only, and the full BG-VMUNet. The results are summarized in Table 5. Post-scan fusion was the dominant contributor: applied alone, it raised Dice to 0.7212 and IoU to 0.6077, close to the full model, and improved Precision and HD95 over the no-guidance model while largely maintaining coverage (Recall 0.8121). Step-size modulation applied alone produced a smaller and more selective effect: compared with the no-boundary-guidance model, it slightly improved Dice from 0.7188 to 0.7198, Precision from 0.7063 to 0.7119, and HD95 from 96.3479 to 94.8510, while IoU slightly decreased from 0.6032 to 0.6016 and Recall decreased from 0.8266 to 0.8153. Thus, step-size modulation alone should be interpreted as a modest boundary-selective adjustment rather than as an independently dominant contributor. Only when the two parts were combined did the model recover Recall to 0.8266 while achieving the best Dice, IoU, and HD95. The two parts therefore appear complementary: post-scan fusion provides the main boundary-adherence gain, whereas step-size modulation contributes a smaller boundary-selective adjustment whose minor coverage trade-off is offset in the full model. Consistent with the statistical analysis above, these component-wise increments are small and should be read as directionally consistent trends under the present sample size rather than as independently significant effects.

**Table 5:** Component-wise ablation of the boundary-guidance mechanism on the AG task.

Model stage	Dice(↑)	IoU(↑)	Precision(↑)	Recall(↑)	HD95(↓)
VMUNet + CoordAtt + modified scan branch	0.7188 ± 0.0630	0.6032 ± 0.0732	0.7063 ± 0.0784	0.8266 ± 0.0371	96.3479 ± 20.1167
+ Δ modulation only	0.7198 ± 0.0535	0.6016 ± 0.0628	0.7119 ± 0.0712	0.8153 ± 0.0283	94.8510 ± 17.2391
+ Post-scan fusion only	0.7212 ± 0.0582	0.6077 ± 0.0566	**0.7137 ± 0.0787**	0.8121 ± 0.0310	95.0211 ± 18.2647
**BG-VMUNet (full)**	**0.7228 ± 0.0504**	**0.6079 ± 0.0592**	0.7111 ± 0.0658	**0.8266 ± 0.0265**	**94.4665 ± 17.8695**

Results are reported as mean ± standard deviation across five-fold cross-validation. Bold text indicates the best value for each metric.

### 3.5 Qualitative assessment in weak-boundary ME-NBI scenarios

**Figure 5:**
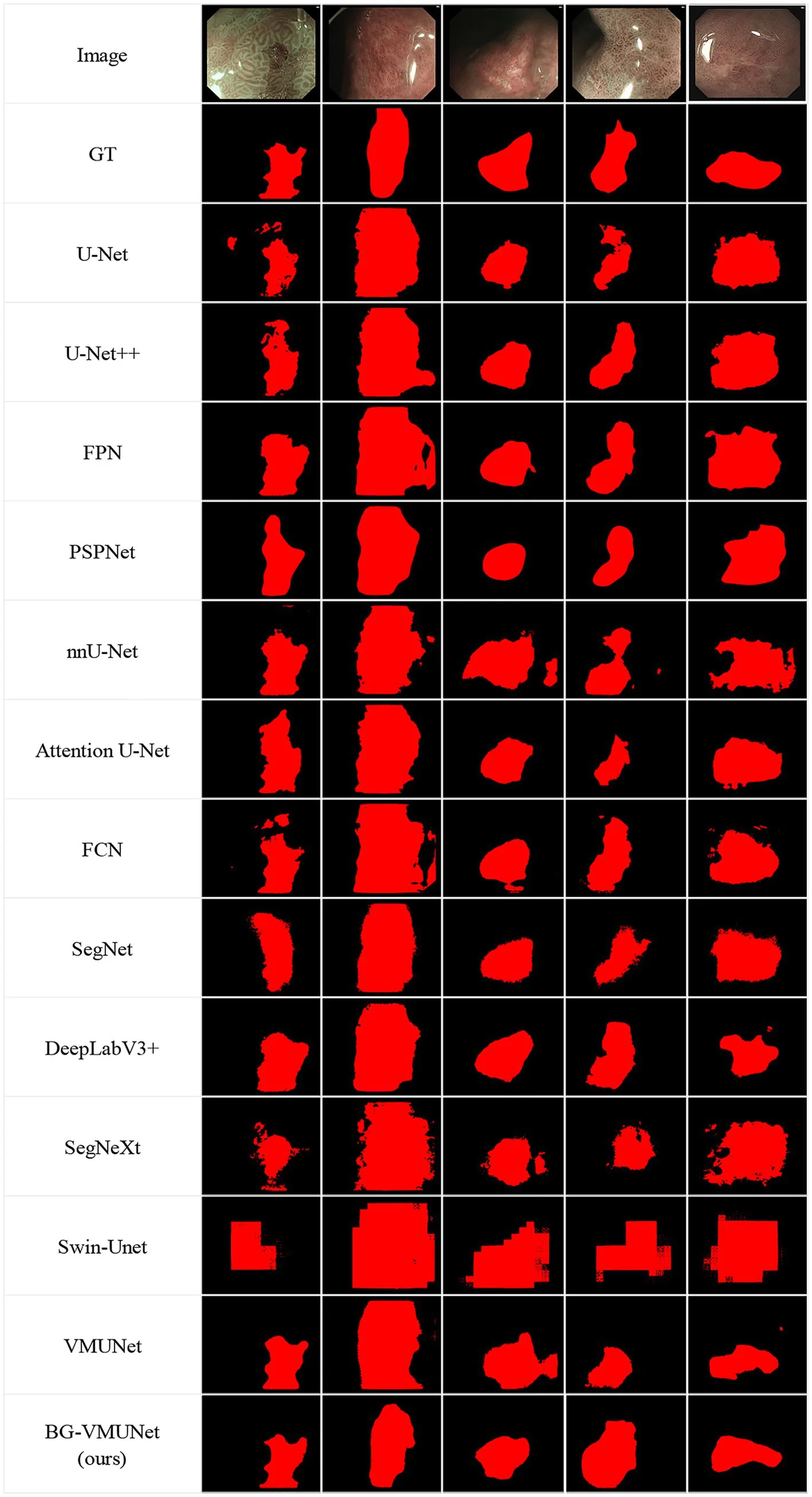
Representative segmentation results for the AG task. The visual examples compare the lesion regions predicted by the model with the corresponding manual annotations, illustrating the model’s ability to delineate atrophic gastritis regions in ME-NBI images.

**Figure 6:**
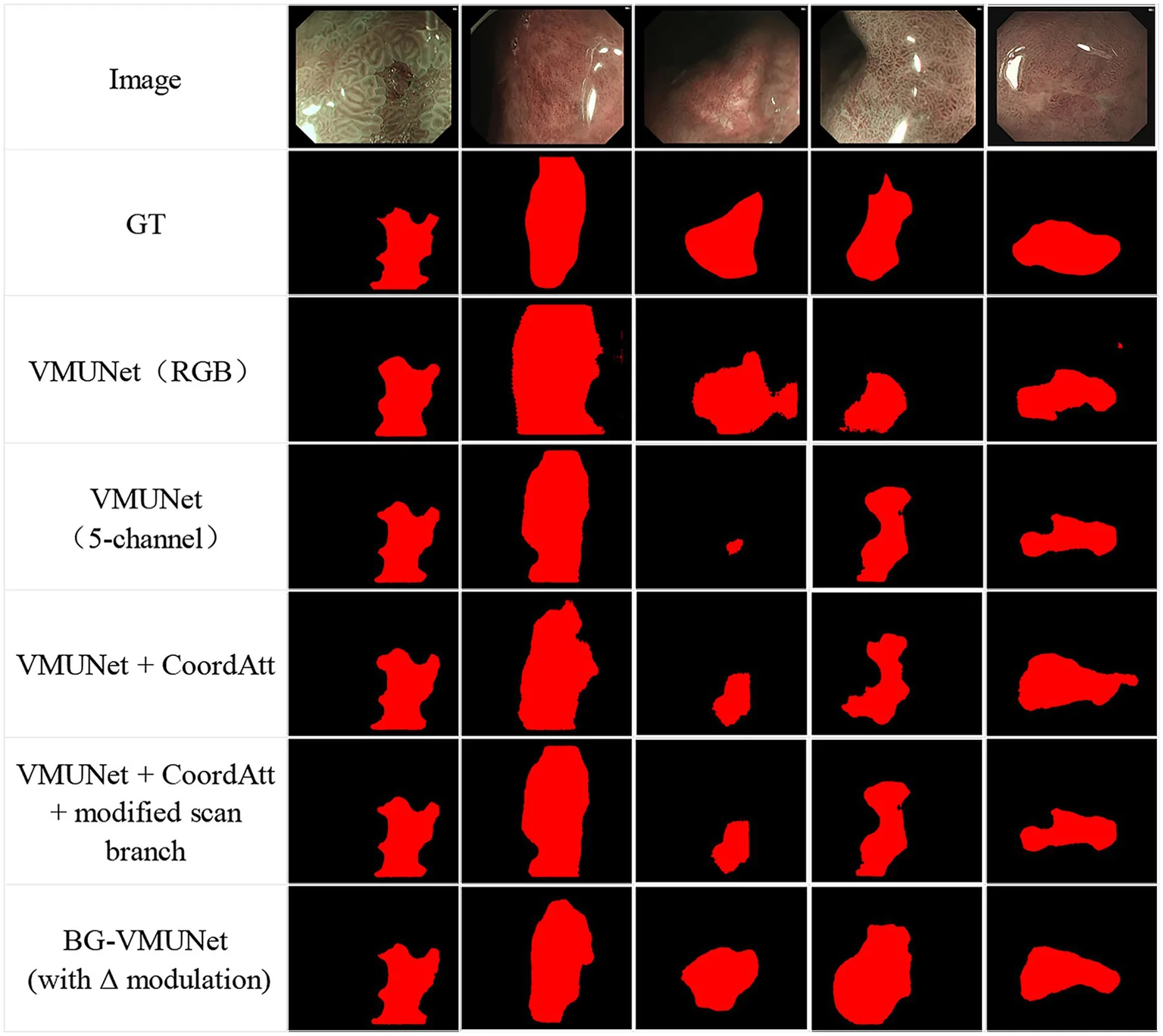
Visual comparison of progressive ablation stages on the AG segmentation task. The examples show the segmentation outputs from the RGB VMUNet baseline, the five-channel input model, the CoordAtt-enhanced model, the modified scan branch model, and the final BG-VMUNet model.

Representative qualitative results are shown in Figures 5, 6. In samples with relatively obvious tonal differences, most models could roughly identify the main lesion area. However, when the surrounding mucosa showed strong texture interference or when the lesion boundary gradually merged with adjacent tissue, baseline models were more likely to produce two types of errors: outward leakage into non-lesion mucosa and incomplete coverage of weakly positive lesion margins. These errors are clinically relevant because they may affect the apparent extent of AG and reduce the consistency of region-level documentation.

Compared with the RGB VMUNet baseline and intermediate configurations, BG-VMUNet produced more stable lesion contours in boundary-ambiguous regions. The final predictions were generally closer to the expert annotations, with better preservation of the main lesion region and fewer unnecessary peripheral responses. In heterogeneous mucosal backgrounds, the model also showed improved suppression of small false-positive areas. These qualitative findings are consistent with the quantitative improvement in Precision and HD95 and support the practical value of incorporating chromatic-textural representation and boundary-guided state-space modeling for ME-NBI AG delineation.

### 3.6 Auxiliary white-light-to-narrow-band augmentation

The auxiliary generative augmentation experiment is summarized in Table 6. When white-light-to-narrow-band synthetic images were added only to the training folds, the Dice of BG-VMUNet increased from 0.7228 to 0.7282, IoU from 0.6079 to 0.6107, and Precision from 0.7111 to 0.7310. HD95 decreased from 94.4665 to 92.2591, whereas Recall slightly decreased from 0.8266 to 0.8162. Because the validation sets consisted exclusively of real ME-NBI images, these findings suggest that style-transfer-based augmentation may improve prediction selectivity and boundary adherence under limited-sample conditions.

**Table 6:** Effect of white-light-to-narrow-band synthetic training data on the performance of the final BG-VMUNet model.

Model	Dice(↑)	IoU(↑)	Precision(↑)	Recall(↑)	HD95(↓)
BG-VMUNet(final, real-only)	0.7228 ± 0.0504	0.6079 ± 0.0592	0.7111 ± 0.0658	**0.8266 ± 0.0265**	94.4665 ± 17.8695
BG-VMUNet(final, + synthetic training data)	**0.7282 ± 0.0511**	**0.6107 ± 0.0604**	**0.7310 ± 0.0781**	0.8162 ± 0.0181	**92.2591 ± 18.3604**

The validation set remains composed only of real ME-NBI images, and the synthetic images are used exclusively as auxiliary training data. Bold text indicates the best value for each metric.

Nevertheless, the slight decrease in Recall indicates that generative augmentation did not uniformly improve all aspects of segmentation behavior. The model became more conservative after synthetic training images were introduced, which may reduce false-positive expansion but may also miss subtle weakly positive regions. Therefore, this experiment should be interpreted as exploratory evidence for improving training diversity rather than as primary evidence for clinical effectiveness. The main conclusions of this study were based on real ME-NBI validation results.

### 3.7 Extended validation on IM lesion segmentation

To examine whether the framework could be applied to another gastric precancerous lesion, BG-VMUNet was further evaluated on IM segmentation. As shown in Table 7, the final model achieved a Dice of 0.7178, IoU of 0.5902, Precision of 0.7305, and HD95 of 96.2425, compared with 0.7096, 0.5797, 0.7020, and 103.5221 for the RGB VMUNet baseline, respectively. Recall decreased from 0.7829 to 0.7648. These results indicate that the final framework improved region overlap, false-positive control, and boundary localization in the IM task, although with a more conservative prediction tendency.

**Table 7:** Extended validation results of BG-VMUNet on the IM segmentation task.

Model	Dice(↑)	IoU(↑)	Precision(↑)	Recall(↑)	HD95(↓)
VMUNet (RGB)	0.7096 ± 0.0283	0.5797 ± 0.0308	0.7020 ± 0.0444	**0.7829 ± 0.0446**	103.5221 ± 7.6453
BG-VMUNet (final)	**0.7178 ± 0.0326**	**0.5902 ± 0.0371**	**0.7305 ± 0.0440**	0.7648 ± 0.0277	**96.2425 ± 17.7436**

The RGB VMUNet baseline and the final BG-VMUNet model are compared to evaluate the transferability of the proposed framework to another type of gastric precancerous lesion. Bold text indicates the best value for each metric.

**Figure 7:**
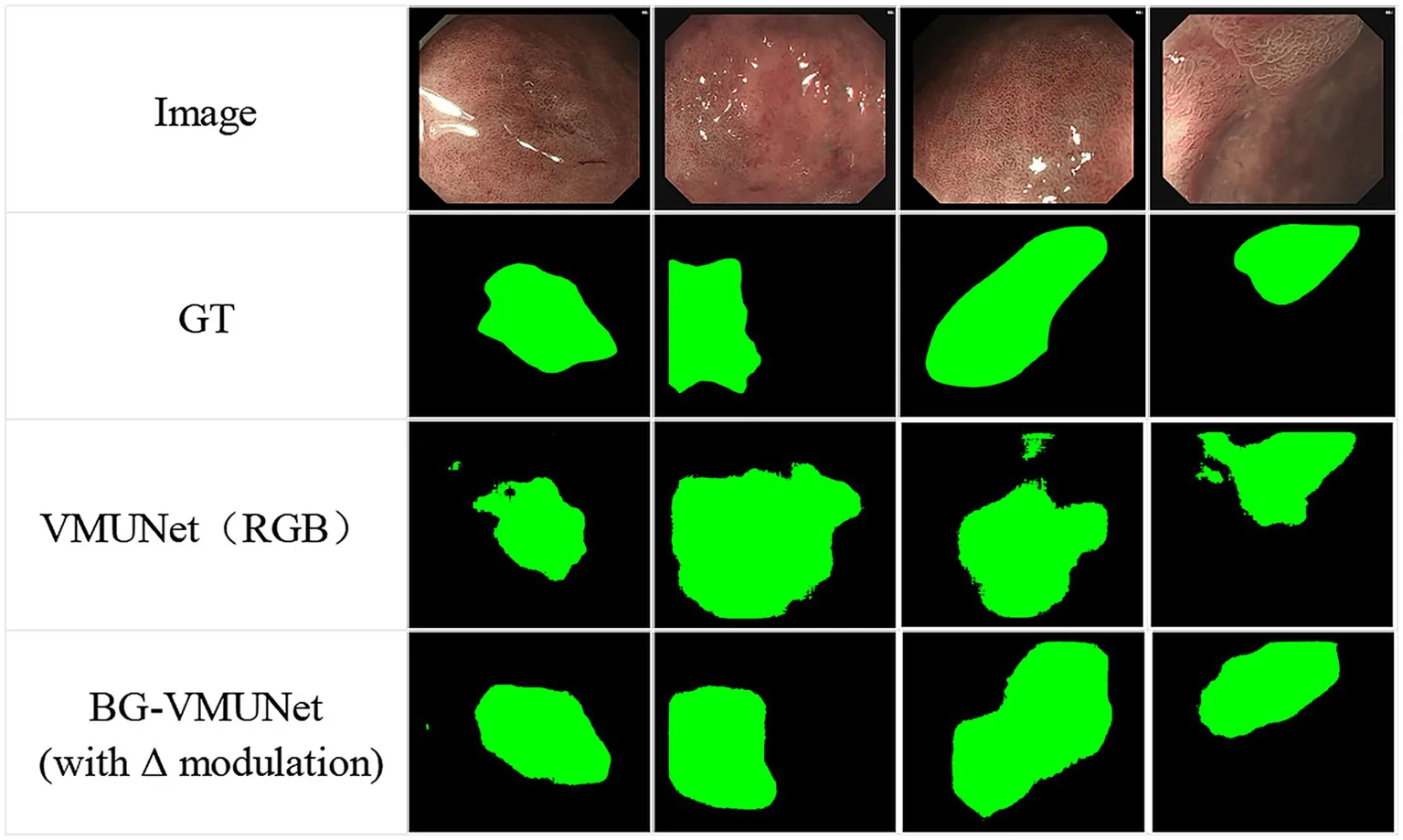
Representative visualization results for the intestinal metaplasia segmentation task. From top to bottom, the examples show the original ME-NBI images, manual annotations, VMUNet baseline predictions, and BG-VMUNet predictions, demonstrating the model’s segmentation performance on IM lesions.

The representative IM visualizations are shown in Figure 7. The RGB VMUNet baseline could roughly identify IM regions, but its predictions sometimes showed rough contours, local overextension, and small residual false-positive regions. In contrast, BG-VMUNet produced smoother and more boundary-adherent contours, especially in samples with heterogeneous texture interference or separated lesion regions. These findings suggest that the design principles used for AG—enhancing chromatic-textural representation, modeling cross-regional mucosal continuity, and strengthening weak-boundary perception—also showed potential utility for IM segmentation. However, because the IM experiment was an extension validation with a limited sample size, the results should be regarded as supportive rather than definitive evidence of generalizability.

## 4 Discussion

This study developed and evaluated a boundary-guided visual state-space segmentation framework for region-level delineation of AG lesions in ME-NBI endoscopic images. The main finding was that BG-VMUNet achieved a better balance among lesion coverage, false-positive control, and boundary localization than the RGB-input VMUNet baseline. In a subject-level paired analysis after aggregating AG validation images within each subject, the improvements over the RGB baseline were statistically significant after Holm correction for the overlap-based metrics, including Dice, IoU, and Precision, whereas the differences in Recall and HD95 were favorable in direction but did not reach significance. This subject-level analysis was used to reduce the influence of correlated images from the same subject while preserving the paired comparison between models. The observed pattern is clinically relevant because the assessment of gastric precancerous lesions depends not only on whether AG is present, but also on how far the lesion extends and how consistently its boundary can be documented.

The clinical motivation of this work differs from conventional object segmentation tasks. AG lesions in ME-NBI images frequently show subtle tonal changes, heterogeneous mucosal texture, and gradual transitions between diseased and surrounding mucosa. These characteristics make the lesion border difficult to define, both for endoscopists and for automated models. The proposed framework was therefore designed around this imaging phenotype. The five-channel representation enhanced color and texture cues through Hue and PIF, visual state-space modeling captured cross-regional mucosal pattern continuity, and boundary-guided modulation strengthened responses around weak transition zones. In this sense, the network was not intended as a generic accumulation of architectural components, but as a problem-oriented segmentation framework for the weak-boundary and heterogeneous-texture characteristics of AG in ME-NBI.

The baseline comparison showed that VMUNet outperformed several classical convolutional, encoder–decoder, transformer-based, and general medical segmentation models under the same RGB input setting. This result suggests that visual state-space modeling is suitable for ME-NBI lesion analysis, where lesion-related information may be spatially distributed rather than confined to a compact region. However, the progressive validation also showed that long-range contextual modeling alone was insufficient. The modified scan branch was intended to strengthen cross-regional contextual propagation and mucosal-pattern continuity, consistent with the higher or maintained Recall at that stage; in ME-NBI images with heterogeneous texture and weak borders, however, stronger propagation can also over-extend into adjacent non-lesion mucosa, consistent with the observed decrease in Precision and increase in HD95. The boundary-guided modulation was designed as a lightweight refinement that emphasizes ambiguous transition zones and suppresses unnecessary peripheral responses, offering a plausible mechanism for the recovery of Precision and HD95 without a loss of Recall. Importantly, the paired analysis showed that the increment of BG-VMUNet over the immediately preceding stage was not statistically significant after Holm correction; we therefore regard small-sample variability as a plausible contributor to this pattern and present it as a directionally favorable trend supported by a plausible mechanism rather than as a proven independent effect.

The qualitative findings further support this interpretation. In representative ME-NBI cases with strong background mucosal texture or gradual lesion transitions, baseline models tended to produce peripheral false-positive responses or incomplete coverage of weakly positive lesion margins. BG-VMUNet generated more stable and boundary-adherent lesion maps, with fewer unnecessary responses in non-lesion mucosa. These visual improvements are important for potential clinical use because an automatically generated mask should be interpretable by endoscopists and should not create misleading impressions of lesion extent. Stable region-level delineation may also facilitate objective lesion-burden estimation, standardized image documentation, and longitudinal comparison during surveillance.

The clinical value of this work should be understood as a preliminary, exploratory step toward decision support rather than autonomous diagnosis. Current guidelines emphasize that the risk associated with AG and IM is closely related to lesion extent, anatomical distribution, and histological severity (2–9). However, most AI studies in gastric precancerous lesions have focused on image-level detection, classification, or risk prediction (14–18). By providing a pixel-level lesion map, the proposed framework may complement these approaches with region-level information. Subject to multicenter external validation and explicit linkage to clinical endpoints, such masks might in future work contribute region-level inputs that could be explored for more objective documentation of suspected AG extent, for selecting representative areas during biopsy, and for risk-oriented surveillance models; these remain potential future directions rather than capabilities demonstrated by the present study. This direction is consistent with the broader goal of digestive imaging AI: transforming image patterns into clinically meaningful outputs for early diagnosis, staging support, and individualized management (19, 20).

The extended validation on IM provided additional supportive evidence. BG-VMUNet improved Dice, IoU, Precision, and HD95 in the IM task compared with the RGB VMUNet baseline, although Recall decreased slightly. This pattern indicates that the framework may generalize to another gastric precancerous lesion scenario, particularly in terms of false-positive control and boundary localization. The decrease in Recall also suggests that the final model tended to be more conservative. For clinical use, this trade-off should be considered carefully: excessive false positives may overestimate lesion extent, whereas missed weakly positive areas may underestimate disease burden. Therefore, future work should explore lesion-specific thresholds, uncertainty visualization, and multi-class AG/IM segmentation to improve the balance between sensitivity and specificity.

The auxiliary white-light-to-narrow-band augmentation experiment showed modest improvements in Dice, Precision, and HD95, but also a slight decrease in Recall. This result suggests that style-transfer-based augmentation may improve model selectivity and boundary adherence by increasing appearance diversity during training. However, synthetic images may also shift the distribution of weak or boundary-ambiguous regions. Therefore, we regard this experiment as exploratory rather than primary evidence of clinical effectiveness. In future studies, generative augmentation should be evaluated with stricter quality control, uncertainty assessment, and external validation before it is incorporated into clinical model development pipelines.

Several limitations should be acknowledged, and the present findings should be regarded as preliminary and exploratory. First, this was a single-center retrospective study based on 137 AG and 87 IM images from 25 subjects, a relatively limited sample size. Although five-fold cross-validation and patient-level allocation were used, the model may still be influenced by center-specific imaging devices, endoscopist habits, and patient characteristics. Second, an independent external test set was not available; the clinical transferability of the model therefore requires validation across multiple centers, devices, operators, and image distributions. Third, the present study evaluated segmentation performance but did not directly link the predicted lesion extent to OLGA/OLGIM stage, histological severity, biopsy strategy, surveillance interval, or gastric cancer risk. Although the diagnosis of AG and IM was supported by clinical records, endoscopic findings, and pathological results, the dataset did not include standardized, lesion-level severity labels with strict one-to-one correspondence to each image; severity grading and severity-stratified subgroup analysis were therefore not performed, and the relatively limited sample size would render such subgroup analysis underpowered. Consequently, the model should not be interpreted as a tool for determining lesion severity, OLGA/OLGIM stage, biopsy strategy, surveillance interval, or gastric cancer risk. Fourth, manual annotation of AG borders remains partly subjective, especially in weak-boundary regions, and a quantitative inter-observer agreement analysis was not performed because expert-consensus masks were used as the reference standard; this residual subjectivity may affect the reference standard. Fifth, the current framework was evaluated on static images rather than full endoscopic videos, and its real-time performance and workflow integration remain to be tested.

Future work should focus on clinical translation. Multicenter external validation is necessary to assess robustness across different ME-NBI systems and clinical environments. Larger datasets should be used to develop joint AG and IM segmentation, multi-class lesion mapping, and uncertainty-aware prediction. More importantly, segmentation outputs should be connected with clinically meaningful endpoints, such as lesion extent ratio, anatomical distribution, histological grading, OLGA/OLGIM staging, targeted biopsy yield, and longitudinal surveillance outcomes. Such integration, together with multicenter external validation, would be required before the model could move beyond technical segmentation performance toward practical support for early recognition and risk stratification of gastric precancerous conditions.

In summary, BG-VMUNet provides a feasible weak-boundary-aware segmentation approach for AG lesion delineation in ME-NBI images. By combining chromatic-textural representation, cross-regional contextual modeling, and boundary-sensitive feature modulation, the framework produced more stable region-level lesion maps in challenging ME-NBI scenarios. Although further external validation and clinical endpoint linkage are required, this study suggests that objective segmentation of gastric precancerous lesions may, in future work, become a useful component of AI-assisted endoscopic assessment, subject to multicenter validation and explicit clinical endpoint linkage.

## Data Availability

The raw data supporting the conclusions of this article will be made available by the authors, without undue reservation.
